# Preliminary study on hazards and critical control points of kokoro, a Nigerian indigenous fermented maize snack

**DOI:** 10.1186/s40064-015-1026-3

**Published:** 2015-06-12

**Authors:** S Oranusi, S O Dahunsi

**Affiliations:** Department of Biological Sciences, Covenant University, Ota, Ogun State Nigeria; Department of Biological Sciences, Landmark University, Omu-Aran, Kwara State Nigeria

**Keywords:** Critical control point, Fermentation, Hazard, Hygiene, Kokoro commercialization, Optimization

## Abstract

The microbial and proximate composition of an indigenous snack from fermented maize was investigated. Critical control points of milling the raw materials, fermentation pH, processing temperature and time intervals during holdings in processing and storage were evaluated with a view to optimizing the product. The mean total aerobic plate count (TAPC) log_10_ values for samples of the finished products range from 2.07 ± 0.50 to 4.36 ± 0.10 cfu/g. Mean fungi count log_10_ was 2.00 ± 0.00 to 3.50 ± 0.50 while mean coliform count 1.04 ± 0.10 log_10_ cfu/g was detected in one of the sales outlets investigated. Bacterial and fungal species were isolated belonging to the genera *Aspergillus*, *Rhizopus*, *Penicillium*, *Fusarium*, *Cephalosporium*, *Alternaria*, *Bacillus*, *Klebsiella*, *Staphylococcus*, *Lactobacillus*, *Pseudomonas*, *Proteus* and *Enterobacter*. The moisture content of the samples ranged from 3.41 to 6.75%; fat content was 19.68 to 32.59%; fiber content was 1.84 to 2.78% while protein ranged from 6.76 to 9.23%. The ash and carbohydrate contents ranged from 1.97 to 2.31% and 49.21 to 61.96%, respectively. Based on the specifications by International Commission for Microbiological Specification for Foods (ICMSF), the TAPC counts of the finished products remained at low levels. However, presence of coliforms could prejudice the hygienic quality of these types of products hence, the need for quality control.

## Background

Kokoro, a fermented maize cake, is produced in Ogun State, Nigeria and mainly in three villages of Imashayi, Joga and Iboro; all in Yewa North Local Government Area. It is widely consumed in the South-western States of Nigeria as snacks to pass time especially between meals. It is also a substitute for *Kwuli*-*kwuli*; a popular groundnut cake. Both snacks complement fried grated and fermented cassava (Gari) when taken in cold water with or without sugar (Braima et al. 2012; Smallstarter 2013).

Kokoro is produced from maize in a 3 day intensive process. The production involves the boiling of whole dried maize for about an hour and then allowing it go through natural fermentation for 24 h. The fermented maize is milled and mixed with wet milled onion (*Allium cepa*) and salt (NaCl) to form paster. The paster is kneaded and fried slightly for 2–4 min, thereafter it is left overnight before being fried a second time for 1–2 min. The finished product is either packaged in cellophane or left open and sold on street trade and/or market places.

The poor quality control and hygiene during preparation affect the taste and texture. There are variations in shelf life from different producers. These actually limit the commercialization of kokoro and HACCP implementation. The public health concerns associated with indigenous production of kokoro arise from the handling practices by individuals with no basic training on food hygiene. The environments are usually unhealthy with domestic animals in and around the processing area, a phenomenon already documented for other food products (Redmond and Griffith 2004a, b, c; Kennedy et al. 2005; Roseman and Kurzynske 2006; Brewer and Rojas 2008; Meysenburg et al. 2014). Water is usually sourced from streams, ponds, wells, few boreholes. Packaging and handling of the products before and during sales to consumers are also source of concern. Like most other ready to eat foods sold on streets, road sides and market places, kokoro is prone and subject to contamination (Oranusi and Olorunfemi 2011; Oranusi and Braide 2012; Pricope et al. 2013; Aung and Chang 2014; Larsen et al. 2014).

Optimization and commercialization of the product for wider acceptability (outside Nigeria) demand standardization of the production procedures and a better understanding of the chemical composition and microbiological problems associated with kokoro production (von Holy and Makhoane 2006; Steyn et al. 2011). The hazard analysis critical control point system is usually employed to identify hazards associated with the different processing stages starting from the raw materials to the finished products and packaging. Such procedure is necessary for monitoring the flow of products, their integrity and the process parameters throughout production also bearing in mind transformations taking place in the state of these products as an effective way of ensuring product safety (Marvin et al. 2009a, b; van de Brug et al. 2014). All these are taken into consideration in this research. The objectives of this preliminary study therefore were to identify the critical points where controls are necessary to prevent hazards as well as to establish measures to produce safe and wholesome products of commercial standard as none is in existence presently.

## Methods

### Description of production area

**Figure 1:**
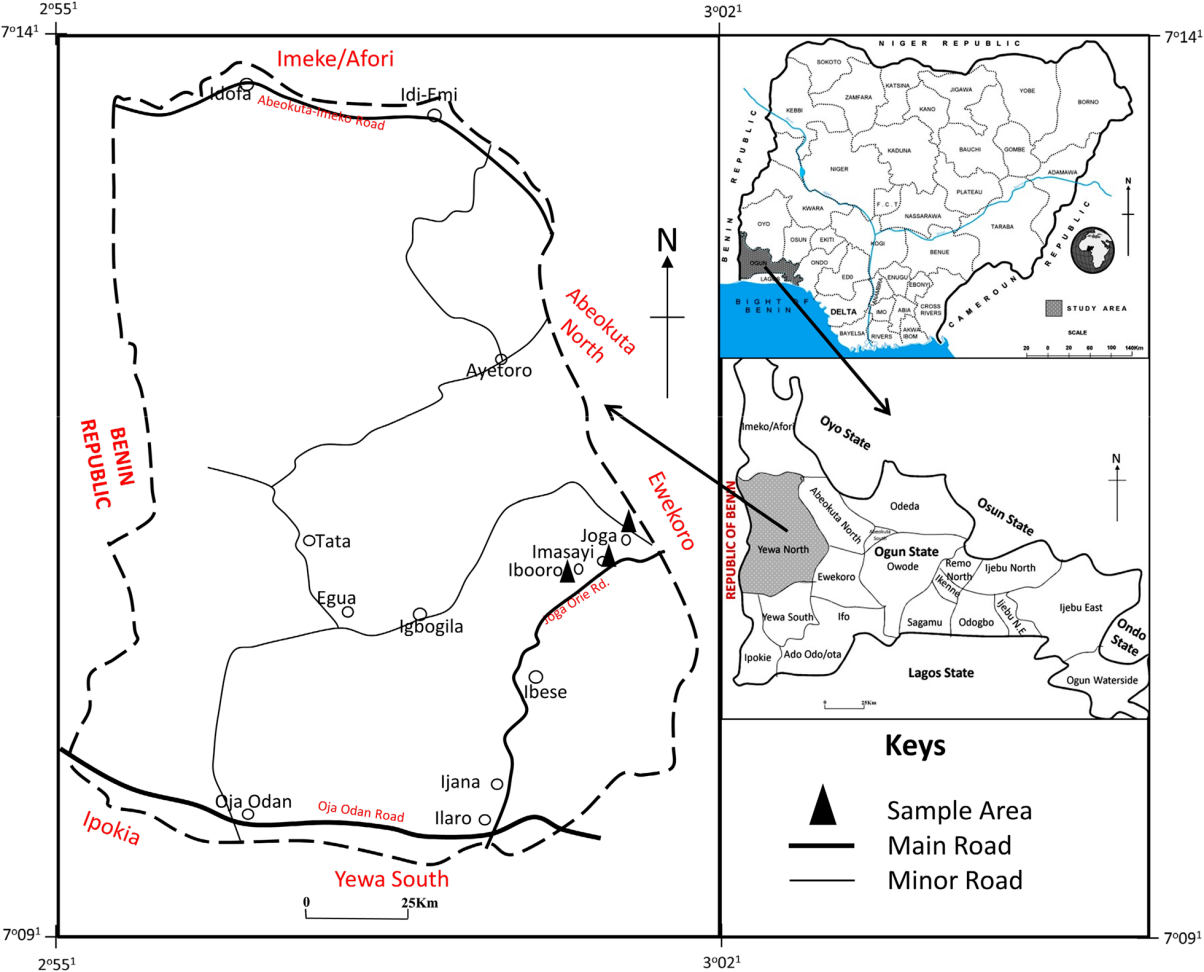
Map of Yewa North showing study area. Source: Ministry of Lands, Surveys and Urban Development, Ogun (2008).

Imashayi, Joga and Iboro communities are places with a very small population in Yewa North Local Government Area situated at 7°14′00″N; 3°02′00″E. It has an area of 2,087 km^2^ (806 sq mi) and a population of 181,826 as at the 2006 census. Imashayi is located at Latitude 7.0833333/7°4′59.9982″, Longitude 3.0833333/3°4′59.9988″ while Joga is positioned between Imashayi and Iboro which occupies Latitude 7.1/7°5′59.9994″, Longitude 3.1/3°6′0.0″. The three communities are agrarian with an expanse of fertile soil. Ilaro is the host community to a Federal polytechnic; Ibese is the host community to Dangote Cement Company (Figure 1). Other cities, towns and places close to these study communities include Joga-Orile and Awaiye. The closest major cities include Abeokuta, Shagamu, Ikorodu and Lagos (GoMapper 2013).

### Sample collection

Sixty samples of kokoro, twenty each from Ibadan (Oyo State), Sango-Ota (Ogun State) and Iyana Ipaja/Oshodi (Lagos State) were purchased from ten different food vendors selected at random in each State. Sampling was done during both rainy and dry seasons. Samples were also collected from local producers at each stage of production and of finished product. Samples purchased from food vendors were transported in cellophane packs wraps as purchased and were kept in ice packs before reaching the laboratory under 24 h. In the same vein, samples from each steps of production were transported in sterile specimen containers held in ice packs. All samples were taken to the laboratory within the same day of collection for microbiological analysis.

### Traditional kokoro production

**Figure 2:**
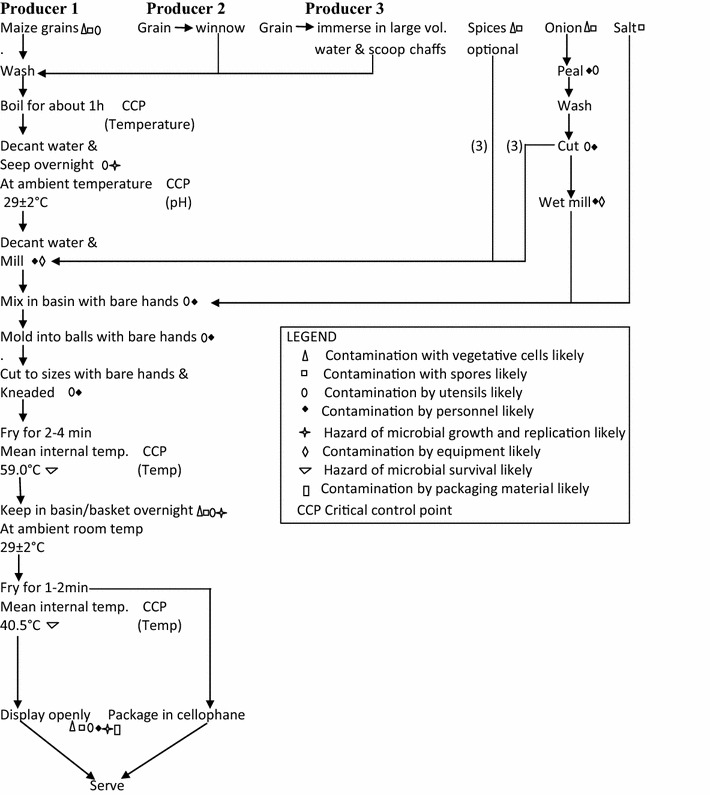
Flow diagram of kokoro preparation and handling.

**Figure 3:**
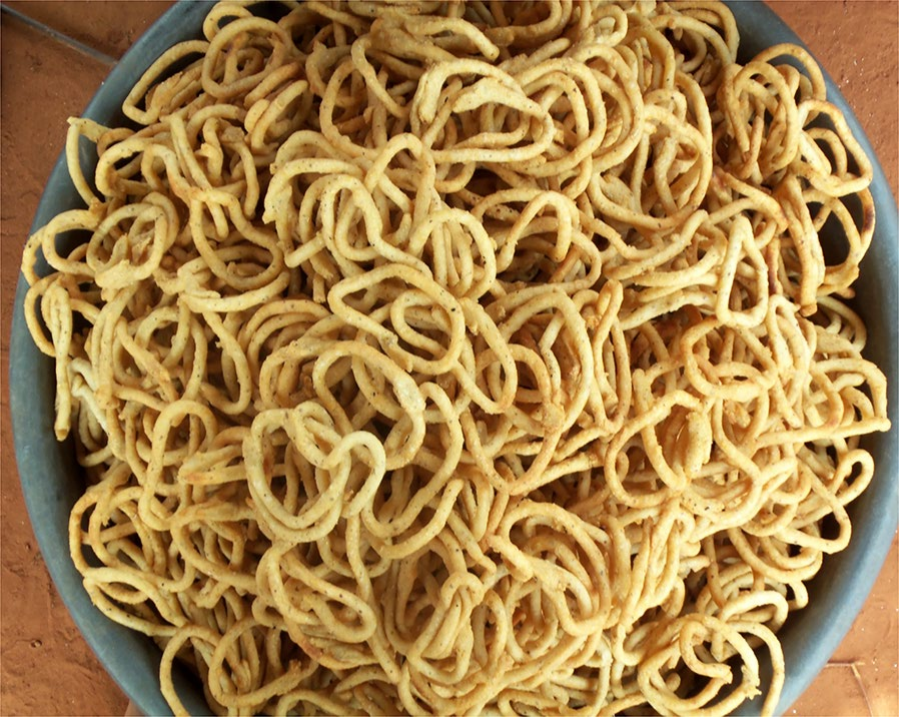
Kokoro after final processing.

Preparation involves the use of maize (*Zea mays*). The grains are washed and boiled for about an hour (depending on the hardness/how dry the grains are). The water used for boiling is decanted and grains steeped overnight to ferment. Fermented grains are sieved from steep water and milled without the addition of water. Salt (NaCl) and wet milled onion (*Allium cepa*) are added and mixed with the milled grains. The mixture is molded into small balls about the size of a medium sized orange. Each ball/mold by experience is estimated to produce a specific quantity of the final product. The small scale producers of kokoro can buy the balls from large scale producers at determined price per dozen to produce the final product at profit after sales. The balls are cut into small sizes and kneaded to produce thin circular or straight snack about 24 cm long, these are slightly fried in ground nut oil for 2–4 min to a light-brown semi-finished product. They are covered in basket/basin overnight before a second (final) frying for 1–2 min to make the product ready for consumption as a light-brown/dark-brown crispy snack. The products are packaged in transparent cellophane or displayed openly for sale (Figures 2, 3).

### Hazard analysis

The hazard analysis and critical control point’s evaluations were conducted on the production protocol of three large scale kokoro producers, one from each of the three communities. Selection was based on the interest of the producers to participate in the exercise in which details of the procedures involved were explained by the researchers during the preliminary visits to the communities. Observations were made of the raw materials used, the personnel involved, the equipment and utensils, the environment, kokoro production and packaging practices to identify sources of actual and potential contaminants. Samples of raw materials and swabs of food contact surfaces were taken at different stages of production (immediately after boiling, during steeping, milling, kneading, after first and second frying and when on display for sales). The thermometer was cleaned in 70% ethanol and dried before use to avoid being a source of contamination to the samples. The analysis also included measuring the temperature–time exposure period and determination of pH during steeping, before frying and after production. The pH was measured using pH and temperature meter (ADWA-AD 1040).

A schematic diagram of kokoro production (Figure 2) was made based on the observations made and questions asked during processing. Previous researchers (Creswell and Clark 2011; Meysenburg et al. 2014) used different types of charts and maps for monitoring the production processes in some food products. Potential sources of contamination, critical control points that needed to be monitored and the likelihood of microbial survival, multiplication or destruction were noted as described by Oranusi et al. (2003).

### Isolation and enumeration of microorganisms

Ten gram of each sample were blended with sterile warring blender and homogenized in 90 mL sterile peptone water. The raw grains where however not blended but soaked for 10–20 min and washed out by vigorous agitation. The resultant homogenate was diluted 10^−2^ to 10^−3^ for the heat treated (boiled and fried) samples and 10^−4^ to 10^−7^ for the raw materials. From the appropriate dilutions, aliquot 0.1 mL was spread plated in triplicate onto different media prepared based on the manufacturer’s instruction. Plate count agar (PCA), eosin methylene blue (EMB) agar and (PDA) potato dextrose agar (all from Biolab, India) were inoculated for total aerobic plate count (TAPC), coliform count and fungal count, respectively. *Bacillus cereus* medium and mannitol salt agar (both from Oxoid, England) were inoculated for isolation of *B. cereus* and *S. aureus* while *Salmonella*-*Shigella* agar (Fluka, Germany) were inoculated after 24 h pre-enrichment of sample homogenate in Selenite F-broth, for isolation of Salmonellae. All inoculated plates were incubated at 37°C for 24–48 h for colony formation and enumeration. Exception to this incubation protocol was PDA plates that were incubated at 29 ± 2°C for 72–120 h and a plate of EMB incubated at 44°C and 24–48 h for faecal coliform organisms. Colonies formed at the expiration of incubation period were counted using digital colony counter (Gallenkamp, England). Counts were expressed as cfu/g of sample. Samples of swabs of food contact surfaces and water were cultured for the presence of coliform organisms, *S. aureus*, *B. cereus* and other organisms concerned with food safety. Characteristic discrete colonies on the different media were isolated, and purified by repeated sub-culturing on Nutrient agar (Oxoid). Pure cultures were stored on agar slants at 4°C for further characterization. For the confirmation of coliform organisms, the method as described by Oranusi et al. (2003) was adopted. Colonies on EMB were inoculated into lactose broth in test tubes with inverted Durham (bell) tubes. Incubation was done for 24–48 h at 37 and 44°C. Gas production and/or color change of dye constituted a positive presumptive test. The broth was inoculated onto EMB plates for 37°C incubation. Typical colonies on EMB appearing bluish black with greenish metallic sheen characteristic of *E. coli* or brown mucoid colonies characteristic of *E. aerogenes* that are Gram negative and non-spore bearing confirmed the presence of coliform organisms. Isolates were stored on agar slants at 4°C for further characterization.

### Identification of isolates

Isolates on slants were purified by repeated sub-culture on nutrient agar. Pure cultures were identified based on standard methods of Jolt et al. (1994). Identification of characteristic bacteria isolates was based on colonial morphology, microscopy and biochemical tests using Biomerieux^®^ Sa API biochemical test kits. Fungal isolates were identified based on their morphology, microscopy and pigmentation on media with reference to standard identification keys and atlas (Tsuneo 2010).

### Proximate analysis of samples

The chemical compositions of the samples were determined according to the procedure outlined by the Association of Official Analytical Chemists (AOAC 1980). The kokoro samples were analyzed for moisture content, carbohydrate, protein, lipid, ash and fiber. Moisture content was obtained by drying samples in moisture dish in an oven at 105°C until constant weights was obtained. For Ash content, pre-dried samples obtained from moisture content analysis were ashed in furnace at 550°C overnight. Crude protein value was obtained from nitrogen which was earlier determined by MicroKjedalh method and by multiplying by 6.25 (conversion factor for nitrogen to protein). Crude fat was obtained by exhaustively extracting 2.0 g of each sample in a Soxhlet apparatus using petroleum ether (b.p. 40–60°C) as the extractant. Determination of crude fibre was done by trichloroacetic acid method (Oladipo and Jadesimi 2012) while carbohydrate content was obtained by difference from the combined percent of moisture, protein, ash and fat from 100 (Nwanze et al. 2006).

### Statistical analyses

The statistical analysis of the data was done using the SPSS 20.0 software for windows (SPSS 2011). The values obtained were confirmed using one-way ANOVA at 0.05 level of significance. Further test on those found to be significant was done using Duncan multiple range tests (DMRT).

#### Significance and impact of study

Kokoro is an ancient snack valued especially within the South western and North central regions of Nigeria comprising of over ten states. It is widely distributed as well. However, this study has shown the various critical and hazard points towards ensuring the safety of this product for consumers. The result of this study is significant as it will be implemented in the bid to commercialize kokoro production in Nigeria as well as ensuring safety and proper hygiene to safeguard public health. It will further contribute to the development of food production database in Nigeria.

## Results and discussion

Figure 1 show the map of the producing towns of kokoro and from where some of the samples (raw materials and finished products) were collected. Figure 2 is a flow diagram showing hazards and critical control points of kokoro production. The figure reveals the activities and attitude of the three different investigated producers in handling the raw material (maize) before real processing commences during which they all eventually follow same pattern throughout the production period. In the figure also, products are shown to be mixed with bare hands and time–temperature exposure is for <5 min and below 60°C. There is also the likelihood of product contamination by vegetative cells and spores as well as microbial survival, growth and multiplication. Sources of potential contamination observed during hazard analysis include dirty food processing environment, animals (goats, chicken and ducks) roaming food processing environment, toilets and bathrooms being located within 50–100 m from processing area and could serve as pools for pathogens proliferation and distribution. Others include children picking and playing with food utensils as well as grinding machine and kneading boards not properly washed and rinsed. The mean microbial load (cfu/g) of samples at different stages of production is presented in Table 1. It shows that milled maize and onion, and water used for processing had significantly (P < 0.05) higher microbial counts compared to other samples. The kokoro products from all the sampling outlets had mean counts of 2–3 log_10_ except however, samples from Ibadan with TAPC in the order of 4 log_10_. Table 2 shows the microbial isolates from the samples and swabs of food contact surfaces. *Bacillus* and *Staphylococcus* spp, Gram negatives—*Pseudomonas*, *Klebsiella* and *Proteus* and moulds—*Aspergillus*, *Rhizopus* and *Penicillium* were the predominant microbial contaminants. The percentage proximate compositions of kokoro from different sales outlets are shown in Table 3. The table reveals significant difference (P < 0.05) for some parameters measured and lower in comparison to whole maize. Moisture is lower in samples from Lagos while kokoro from Ibadan gave a lower value for carbohydrate and higher value for fat when compared to other samples.

**Table 1:** Mean microbial counts log_10_ (cfu/g) of samples at different stages of production

Sample types	pH	Mean microbial count		
		TAPC	Coliform count	Fungal count
Producer 1				
Water for processing	6.7	6.32 ± 0.2	6.04 ± 0.7	–
Raw maize	6.4	4.85 ± 0.4	3.53 ± 0.1	4.55 ± 0.6
Boiled maize	6.0	1.39 ± 0.2	–	1.07 ± 0.5
Fermented maize	5.8	7.14 ± 0.6	2.00 ± 0.0	4.84 ± 0.5
Steep water after fermentation	5.8	7.25 ± 0.5	2.07 ± 0.9	4.79 ± 0.2
Milled maize	6.0	7.32 ± 0.3	1.00 ± 0.1	7.36 ± 0.1
Grinded onion	6.4	6.49 ± 0.1	5.27 ± 0.8	4.74 ± 0.8
Milled and kneaded maize	6.2	7.57 ± 1.0	5.30 ± 0.2	7.53 ± 0.4
Product after first frying	NA	1.77 ± 0.6	–	1.00 ± 0.0
1st fried product left overnight	NA	3.91 ± 0.3	–	3.36 ± 0.1
Product after final frying	6.1	2.07 ± 0.5	–	2.30 ± 0.1
Product bought from Lagos^a^	6.4	3.00 ± 0.0	1.04 ± 0.1	2.07 ± 0.5
Product bought from Ibadan^a^	6.0	4.36 ± 0.1	–	3.50 ± 0.5
Product bought from Ota^a^	5.9	3.41 ± 0.4	–	2.14 ± 0.6
Producer 2				
Water for processing	6.8	5.07 ± 0.2	3.20 ± 0.4	–
Raw maize	6.4	5.62 ± 0.3	–	5.81 ± 0.7
Boiled maize	5.9	2.04 ± 0.1	–	3.00 ± 0.0
Fermented maize	5.4	8.85 ± 0.6	1.00 ± 0.0	4.60 ± 0.2
Steep water after fermentation	5.4	8.93 ± 1.0	1.00 ± 0.0	4.66 ± 0.3
Milled maize	6.2	9.11 ± 0.5	3.32 ± 0.2	6.34 ± 0.4
Grinded onion	6.5	5.34 ± 0.2	–	4.78 ± 0.5
Milled and kneaded maize	6.1	9.14 ± 0.6	3.30 ± 0.1	6.64 ± 0.2
Product after first frying	NA	2.85 ± 0.1	–	1.34 ± 0.3
1st fried product left overnight	NA	2.79 ± 0.4	–	2.32 ± 0.2
Product after final frying	6.0	2.63 ± 0.3	–	2.30 ± 0.1
Producer 3				
Water for processing	6.7	4.63 ± 0.3	4.34 ± 0.2	–
Raw maize	6.5	4.93 ± 0.4	–	4.55 ± 0.6
Boiled maize	6.1	–	–	–
Fermented maize	5.7	7.25 ± 0.6	4.07 ± 0.9	3.30 ± 0.1
Steep water after fermentation	5.7	9.74 ± 0.8	4.30 ± 0.1	5.49 ± 0.2
Milled maize	6.3	7.61 ± 0.2	3.90 ± 0.3	7.17 ± 0.6
Grinded onion	NA	NA	NA	NA
Milled and kneaded maize	6.3	6.94 ± 0.7	4.79 ± 0.0	4.96 ± 0.8
Product after first frying	NA	2.50 ± 0.5	–	–
1st fried product left overnight	NA	3.61 ± 0.3	–	2.07 ± 0.2
Product after final frying	6.2	2.04 ± 0.1	–	2.00 ± 0.00

Producer = local producers of kokoro at the three sampling communities.

*NA* not applicable, *TAPC* total aerobic plate count.

^a^Samples purchased from food vendors in other sales outlets.

**Table 2:** Microorganisms isolated from snack and food contact surfaces

Sample types	Microbial species isolated
Water for processing	*Bacillus*, *Enterobacter*
Raw maize	*Bacillus*, *Klebsiella*, *Aspergillus*, *Rhizopus*
Boiled maize	*Bacillus*
Fermented maize	*Bacillus*, *Pseudomonas*, *Lactobacillus*, *Staphylococcus*, *Aspergillus*, *Rhizopus*
Steep water after fermentation	*Bacillus*, *Staphylococcus*, *Lactobacillus*, *Pseudomonas*, *Aspergillus*, *Rhizopus*
Milled maize	*Proteus*, *Pseudomonas*, *Bacillus*, *Staphylococcus*, *Lactobacillus*, *Cephalosporium*, *Aspergillus*, *Rhizopus*
Grinded onion	*Klebsiella*, *Proteus*, *Staphylococcus*, *Cephalosporium*, *Aspergillus*, *Rhizopus*
Milled and kneaded maize	*Klebsiella*, *Proteus*, *Staphylococcus*, *Pseudomonas*, *Aspergillus*, *Cephalosporium*, *Penicillium*
Product after first frying	*Bacillus*, *Rhizopus*
1st fried product left overnight	*Bacillus*, *Rhizopus*
Product after final frying	*Bacillus*, *Rhizopus*
Product purchased from Lagos	*Bacillus*, *Klebsiella*, Mucor
Product purchased from Ibadan	*Pseudomonas*, *Bacillus*, *Aspergillus*, *Alternaria*
Product from Sango-Ota	*Staphylococcus*, *Bacillus*, *Penicillium*, *Fusarium*
Swab of bowls for mixing and holding samples	*Pseudomonas*, *Bacillus*, *Aspergillus*, *Penicillium*
Swabs of kneading board	*Bacillus*, *Staphylococcus*, *Aspergillus*
Swabs of grinding machine	*Bacillus*, *Pseudomonas*, *Proteus*, *Staphylococcus*, *Klebsiella*, *Aspergillus*, *Penicillium*, *Cephalosporium*
Hand swabs of personnel	*Bacillus*, *Staphylococcus*, *Aspergillus*

**Table 3:** Percentage proximate compositions (mean values) of products from different sales outlets

Percentage proximate analysis (% w/w)	Sample collection spot				Whole maize (Oke 1965)
	Lagos	Study areas	Ibadan	Sango-Ota	
Moisture	3.41 ± 0.02	6.65 ± 2.00	6.50 ± 2.01	6.75 ± 1.03	NG
Fat (ether extract)	24.17 ± 2.01	23.58 ± 3.01	32.59 ± 0.02	19.68 ± 3.02	4.1 ± 1.02
Crude fibre	1.89 ± 1.02	1.84 ± 0.01	2.78 ± 0.01	2.28 ± 0.01	1.3 ± 0.02
Crude protein	6.80 ± 1.03	9.23 ± 2.03	6.76 ± 2.01	7.07 ± 1.03	11.8 ± 2.04
Ash	1.97 ± 0.01	2.31 ± 0.02	2.16 ± 0.01	2.26 ± 0.12	3.7 ± 0.01
Carbohydrate	61.76 ± 3.05	56.38 ± 1.02	49.21 ± 2.02	61.96 ± 0.22	82.6 ± 3.02

*NG* value not given.

Grains used in the production of kokoro are sourced from the open market and are not of any specific standard in terms of microbial load, moisture content and storage conditions. The major criteria for purchase are cost and easy availability. Quality control and commercialization of this product will demand adequate traceability of the raw materials (Wang and Li 2006; Karlsen et al. 2013). Washing of the grain will likely reduce the microbial load by one to two log but this largely depend on the quality of water used. Water is also an essential raw material and a medium needed to sanitize the food contact surfaces (equipment, utensils, and personnel) and the environment. Poor quality water will give a bad product irrespective of the quality of the other input materials (Ruini et al. 2013).

Boiling of the grains is a heat treatment so also is frying. Heat treatment not only improves the product’s taste, smell, appearance and digestibility, it also reduces the number of microorganisms, improves quality and the overall safety of food. It is thus a critical control point (CCP), (Oranusi et al. 2003). Boiling for about 1 h will most likely destroy vegetative cells. The mean internal temperature of 59.0 and 40.5°C which the product attained for a period of 1–4 min may not be sufficient to achieve reductions of spores and thermo-tolerant microorganisms. Kokoro therefore cannot be said to be completely safe because these temperatures are relied upon by the CCP for safety of the product.

Steeping of grain overnight allows for fermentation, consequently pH reduction. Fermentation improves the keeping quality and safety of fermented foods, pH is thus a CCP. The steeping process also allows for the softening of the maize kernel, improves milling and product quality. It also reduces cooking and food preparations period. It increases some nutrients (Odunfa 1994; Afoakwa et al. 2007), however, via leaching some other nutrients loss are often inevitable (Osungbaro 2009; Aminigo and Akingbala 2004). Steeping/fermentation can also encourage growth and multiplication of contaminants and selection for acidophiles (Justé et al. 2011). Fermentation of grain for kokoro production is by mixed microflora from the environment and quality control is lacking in the traditional kokoro production.

Milling and mixing processes are procedures aimed at producing good quality products. However, these processes expose the product to possible contamination specifically when the milling machine and kneading board are not properly washed and rinsed to remove previous food particles. Bare hands were also used in the transfer of grains and onion to be grinded and in the mixing and kneading of the materials.

The high TAPC and coliform counts recorded for water used for the processing of kokoro by all three producers is higher than the standard specification (SON 2007; WHO 2011). The water is therefore of poor quality. Water used for kokoro production was fetched from the drums with cups and plates often picked from the floor, contamination could have resulted from the use of these utensils. The primary source of the water, river, pond and borehole could equally be contaminated.

The maize was boiled and the microbial load after boiling was low prior to fermentation and milling. The high microbial load recorded for the milled maize could be explained to have originated either from growth and multiplication of contaminants during fermentation, additions from the grinding machine, wet milled onion, the water or combination of these sources. It could also be a reflection of the level of exposures and the handling processes.

### Microbial quality

*Bacillus* spp are spore formers and are common environmental contaminants. They have been implicated in numerous ready to eat foods and snacks (Umoh et al. 2004; Oranusi and Olorunfemi 2011; Oranusi and Braide 2012; Lesley et al. 2013). Moulds, *Aspergillus*, *Penicillium* and *Fusarium* form resistant spores which are tolerant to low pH. They are contaminants in the environment; their presence in the final product can thus be explained. Some strains of these moulds are known to produce deleterious mycotoxin under favourable conditions and are good food spoilage agents. The presence of these moulds in kokoro must therefore be controlled specifically by prompt product consumption and proper storage.

*Staphylococcus aureus*, *Pseudomonas* and *Klebsiella* spp are known to survive extreme environmental conditions like high temperature, pH etc. They are commonly implicated in food as contaminants and have been associated with food borne disease and spoilage. *S. aureus* a common human normal flora could have been introduced via the personnel and this is of serious concern because the organism has been implicated in food poisoning (Hennekinne et al. 2012). Staphylococcal food poisoning (SFP) is of serious concern in the food industry and has been reported as a major food-borne disease affecting several thousands of people annually throughout the world (Hazariwala et al. 2002; Asao et al. 2003; Hennekinne et al. 2012; Ji-Yeon et al. 2013; Tallent et al. 2013). Another major source of contamination could be the packaging materials (cellophane) which have been reported to be opened for use by blowing air into it with the bare mouth thereby introducing germs (Oranusi and Olorunfemi 2011).

The proximate composition of kokoro reported in this work with the exception of percentage fat, fiber and ash contents is lower than the composition reported for whole maize grain (Oke 1965). This could be attributed to losses in nutritional composition due to leaching often associated with steeping of grains for fermentation (Akobundu and Hoskins 1982; Osungbaro 2009; Sanni et al. 2001). The moisture content reported for the samples from Lagos are significantly (P < 0.05) lower than others. Low moisture content is a reflection of low water activity (a^w^) which in turn reduces microbial proliferation rate and a necessary factor in the extension of shelf life and effective storage of products. Therefore, the samples from Lagos can be said to be more shelf stable than others. The fat content (ether extract) of kokoro samples is significantly (P < 0.05) higher than the whole grain. This could be explained by the use of groundnut oil in the frying of kokoro. Samples from Ibadan had higher (P < 0.05) fat content when compared to other samples. Similarly, the carbohydrate content of samples from Ibadan was significantly (P < 0.05) lower compared to other samples all of which have lower values than whole maize grain. Losses associated with leaching during steeping of grains for fermentation could be responsible for the low contents of carbohydrate in kokoro. Fat and carbohydrate are energy dense content of food, kokoro is thus a rich source of energy. The percentage of crude protein of the samples from Imashayi was higher than the other samples but lower than the content of whole maize grain. The ash content of the kokoro samples was higher than the content for whole grains. This could be attributed to the onion and salt content of kokoro.

In conclusion, the samples examined in this study can be said to be nutritionally rich (contain adequate basic food nutrients). Commercialization may however demand some levels of fortification to make the product nutritionally richer (addition of vitamin A and other approved nutrients). There should be quality control of the production process with the development of specific starter culture, standardization of temperature, time, pH and microbial loads during processing. There should also be appropriate packaging quality control. The personnel involved in the production should be educated on the hazards and critical control points of kokoro production. Good manufacturing practices will aid in making this product safe, wholesome and a delight for consumers.

## References

[CR1] Afoakwa EO, Sefa-Dedeh S, Simpson BS, Sakyi-Dawson AE, Asomaning J (2007). Influence of spontaneous fermentation on some quality characteristics of maize-based cowpea-fortified nixtamalized foods. Af J Food Agric Nutr Dev.

[CR2] Akobundu ENT, Hoskins FH (1982). Protein losses in traditional agidi paste. J Food Sci.

[CR3] Aminigo ER, Akingbala JO (2004). Nutritive composition of Ogi fortified with Okra seed meal. J Appl Sci Environ Manag.

[CR4] AOAC (1980) Official methods of analysis of the association of official analytical chemists. 13th edn. AOAC, Washington DC

[CR5] Asao T, Kumeda Y, Kawai T, Shibata T, Oda H, Haruki K (2003). An extensive outbreak of staphylococcal food poisoning due to low-fat milk in Japan: estimation of enterotoxin A in the incriminated milk and powdered skim milk. Epidemiol Inf.

[CR6] Aung MM, Chang YS (2014). Traceability in a food supply chain: safety and quality perspectives. Food Contr.

[CR7] Braima J, Richardson O, Adebayo A, Sylvanus F, Bussie M, Lateef S et al (2012) Producing Gari from Cassava an illustrated guide for smallholder cassava processors. A production by the International Institute of Tropical Agriculture (IITA), with support from the United States Agency for International Development (USAID) and the Technical Centre for Agricultural and Rural Cooperation (CTA)

[CR8] Brewer MS, Rojas M (2008). Consumer attitudes toward issues in food safety. J Food Saf.

[CR9] Creswell JW, Clark VL (2011). Designing and conducting mixed methods research.

[CR11] GoMapper (2013) http://www.gomapper.com. Accessed 25 Oct 2013

[CR12] Hazariwala A, Sanders Q, Hudson CR, Hofacre C, Thayer SG, Maurer JJ (2002). Distribution of staphylococcal enterotoxin genes among *Staphylococcus aureus* isolates from poultry and humans with invasive staphylococcal disease. Av Dis.

[CR13] Hennekinne J, De Buyser M, Dragacci S (2012). *Staphylococcus aureus* and its food poisoning toxins: characterization and outbreak investigation. FEMS Microbiol Rev.

[CR15] Ji-Yeon H, Chang G, Bing S, Kwon K, Lee H, Kim S (2013). A foodborne outbreak of *Staphylococcus aureus* associated with fried chicken in Republic of Korea. J Microbiol Biotech.

[CR16] Jolt JG, Krieg NR, Sneath PHA, Stanley JT, Williams ST (1994). Bergey’s manual of systematic bacteriology.

[CR17] Justé A, Malfliet S, Lenaerts M, De Cooman L, Aerts G, Willems KA (2011). Microflora during malting of barley: overview and impact on malt quality. Brew Sci.

[CR18] Karlsen KM, Dreyer B, Olsen P, Elvevoll EO (2013). Literature review: does a common theoretical framework to implement food traceability exist?. Food Contr.

[CR19] Kennedy J, Jackson V, Cowan C, Blair I, McDowell D, Bolton D (2005). Consumer food safety knowledge. Segmentation of Irish home food preparers based on food safety knowledge and practice. Br Food J.

[CR20] Larsen MH, Dalmasso M, Ingmer H, Langsrud S, Malakauskas M, Mader A (2014). Persistence of foodborne pathogens and their control in primary and secondary food production chains. Food Contr.

[CR21] Lesley MB, Velnetti L, Yousr AN, Kasing A, Samuel L (2013). Presence of *Bacillus cereus* s.l. from ready-to-eat cereals (RTE) products in Sarawak. Int Food Res J.

[CR22] Marvin HJP, Kleter GA, Frewer LJ, Cope S, Wentholt MTA, Rowe G (2009). A working procedure for identifying emerging food safety issues at an early stage: implications for European and international risk management practices. Food Contr.

[CR23] Marvin HJP, Kleter GA, Prandini A, Dekkers S, Bolton DJ (2009). Early identification systems for emerging foodborne hazards. Food Chem Toxicol.

[CR24] Meysenburg R, Albrecht JA, Litchfield R, Ritter-Gooder PK (2014). Food safety knowledge, practices and beliefs of primary food preparers in families with young children. A mixed methods study. Appetite.

[CR25] Nwanze PI, Jatto W, Oranusi S, Josiah SJ (2006). Proximate analysis of *Lentinus squarrosulus* (Mont.) Singer and *Psathyrella atroumbonata* Pegler. *Af*. J Biotech.

[CR26] Odunfa SA (1994). Development of starter cultures for nutritional enrichment of fermented cereals gruel. J Appl Bacteriol.

[CR27] Oke OL (1965) Chemical studies on some Nigerian cereals. http://www.aaccnet.org/publications/cc/backissues/1965/Documents/Chem42_299.pdf. Accessed 25 Oct 2013

[CR28] Oladipo IC, Jadesimi PD (2012). Microbiological analysis and nutritional evaluation of West African soft cheese (*wara*) produced with different preservatives. Am J Food Nutr.

[CR29] Oranusi US, Braide W (2012). A study of microbial safety of ready-to-eat foods vended on highways: Onitsha-Owerri, South east Nigeria. Int Res J Microbiol.

[CR30] Oranusi S, Olorunfemi OJ (2011). Microbiological safety evaluation of snacks sold in fast food shops in Ota, Ogun state, Nigeria. Int J Agric Food Sci.

[CR31] Oranusi SU, Umoh VJ, Kwaga JKP (2003). Hazard and critical points of kunun-zaki, a non-alcoholic beverage in Northern Nigeria. Food Microbiol.

[CR32] Osungbaro TO (2009). Physical and nutritive properties of fermented cereal foods. Af J Food Sci.

[CR33] Pricope L, Nicolau A, Wagner M, Rychli K (2013). The effect of sub-lethal concentrations of benzalkonium chloride on invasiveness and intracellular proliferation of *Listeria monocytogenes*. Food Contr.

[CR34] Redmond EC, Griffith CJ (2004). Consumer attitudes and perceptions towards microbial food safety in the domestic kitchen. J Food Saf.

[CR35] Redmond EC, Griffith CJ (2004). Consumer perceptions of food safety risk, control and responsibility. Appetite.

[CR36] Redmond EC, Griffith CJ (2004). Microbiological and observational analysis of cross contamination risks during domestic food preparation. Br Food J.

[CR38] Roseman M, Kurzynske J (2006). Food safety perceptions and behaviors of Kentucky consumers. J Food Prod.

[CR39] Ruini L, Marino M, Pignatelli S, Laio F, Ridolfi L (2013). Water footprint of a large-sized food company: the case of Barilla pasta production. Water Res Ind.

[CR40] Smallstarter (2013) Gari and Cassava production—a small business that can change your life! www.Smallstarter.com/brows-idea/agribusiness-andfood/author/574-smallstarterthinkthank. Accessed 23 Oct 2013

[CR1001] Sanni AI, Asiedu M, Ayernor GS (2011). Influence of processing conditions on the nutritive value of Ogi-Baba, a Nigerian fermented sorghum gruel. Plant Foods Hum Nutr.

[CR41] SPSS (2011) IBM SPSS software for Windows version 20.0, SPSS Inc., Chicago, IL

[CR42] Standards Organization of Nigeria (SON) (2007) Nigerian standard for drinking water

[CR43] Steyn NP, Labadaries D, Nel JH (2011). Factors which influence the consumption of street foods and fast foods in South Africa—a national survey. Nutr J.

[CR44] Tallent SM, DeGrasse JA, Wang N, Mattis DM, Kranz DM (2013). Novel platform for the detection of Staphylococcus aureus enterotoxin B in foods. Appl Environ Microbiol.

[CR46] Tsuneo W (2010). Pictorial atlas of soil and seed fungi: morphologies of cultural fungi and key to species.

[CR47] Umoh VJ, Oranusi SU, Kwaga JKP (2004). The public health significance of pathogens isolated from Kunun-Zaki sold in retail outlets in Zaria, Nigeria. Niger Food J.

[CR48] van de Brug FJ, Luijckx BL, Cnossen HJ, Houben GF (2014). Early signals for emerging food safety risks: from past cases to future identification. Food Contr.

[CR49] von Holy A, Makhoane FM (2006). Improving street food vending in South Africa: achievements and lessons learned. Int J Food Microbiol.

[CR50] Wang X, Li D (2006) Value added on food traceability: a supply chain management approach. In IEEE conference on service operations and logistics, and informatics (SOLI 2006). The Institute of Electrical and Electronics Engineers (IEEE), Shanghai, China, pp. 493–498

[CR51] WHO (2011) Guidelines for drinking-water quality. 4th edn 2006 census Yewa North figure. http://en.wikipedia.org/wiki/Yewa_North. Accessed 31 Oct 2013

